# Origin and Alteration of Organic Matter in Termite Mounds from Different Feeding Guilds of the Amazon Rainforests

**DOI:** 10.1371/journal.pone.0123790

**Published:** 2015-04-24

**Authors:** Nina Siebers, Christopher Martius, Kai-Uwe Eckhardt, Marcos V. B. Garcia, Peter Leinweber, Wulf Amelung

**Affiliations:** 1 Institute of Crop Science and Resource Conservation (INRES), Soil Science and Soil Ecology, University of Bonn, Nussallee 13, 53115, Bonn, Germany; 2 Center of Development Research (ZEF), University of Bonn, Walter-Flex-Straße 3, 53113, Bonn, Germany; 3 Soil Science, University of Rostock, Justus-von-Liebig Weg 6, 18051, Rostock, Germany; 4 Embrapa Amazônia Ocidental, Rodovia AM-010, Km 29, (Estrada Manaus/Itacoatiara), Caixa Postal 319, CEP: 69010–970, Manaus/AM, Brasil; University of California Davis, UNITED STATES

## Abstract

The impact of termites on nutrient cycling and tropical soil formation depends on their feeding habits and related material transformation. The identification of food sources, however, is difficult, because they are variable and changed by termite activity and nest construction. Here, we related the sources and alteration of organic matter in nests from seven different termite genera and feeding habits in the Terra Firme rainforests to the properties of potential food sources soil, wood, and microepiphytes. Chemical analyses comprised isotopic composition of C and N, cellulosic (CPS), non-cellulosic (NCPS), and N-containing saccharides, and molecular composition screening using pyrolysis-field ionization mass spectrometry (Py-FIMS). The isotopic analysis revealed higher soil δ^13^C (-27.4‰) and δ^15^N (6.6‰) values in nests of wood feeding *Nasutitermes* and *Cornitermes* than in wood samples (δ^13^C = -29.1‰, δ^15^N = 3.4‰), reflecting stable-isotope enrichment with organic matter alterations during or after nest construction. This result was confirmed by elevated NCPS:CPS ratios, indicating a preferential cellulose decomposition in the nests. High portions of muramic acid (MurAc) pointed to the participation of bacteria in the transformation processes. Non-metric multidimensional scaling (NMDS) revealed increasing geophagy in the sequence *Termes* < *Embiratermes* < *Anoplotermes* and increasing xylophagy for *Cornitermes* < *Nasutitermes*, and that the nest material of *Constrictotermes* was similar to the microepiphytes sample, confirming the report that *Constrictotermes* belongs to the microepiphyte-feeders. We therewith document that nest chemistry of rainforest termites shows variations and evidence of modification by microbial processes, but nevertheless it primarily reflects the trophic niches of the constructors.

## Introduction

Termites (Isoptera) play a major role in the functioning of tropical ecosystems, as they contribute to nutrient cycling and soil-forming processes [1–4]. Overall, tropical termites may consume up to half of the annual litter production [5] and up to 90% of dead wood [6]. Therefore, detailed knowledge about feeding habits is crucial for understanding the role of termite diversity on organic matter transformations and the biogeochemistry of the ecosystem. Especially in tropical rainforests termites significantly contribute to organic matter breakdown and thus nutrient cycling, also because termites are essential for initial stages of litter layer decomposition processes [7]. Depending on the food source, the termites may be classified into three principal feeding guilds, mainly i) wood-feeders (xylophagous termites), ii) soil-feeders consuming organic residues in the soil (humivorous termites), and iii) termites feeding on both wood and organic residues (soil/wood-interface feeders, [3]). Besides, there are niche-feeders like *Constrictotermes cavifrons*, which have been shown to feed on microepiphytes [8, 9]. Frequently, however, a direct proof for the food source is missing, due to the high variability of potential food sources and restriction in current methods to identify specific food sources [10]. Therefore, a complementary approach using state-of-the-art techniques in conjunction with well-established chemical analyses is necessary to unambiguously identify food sources used by different termite genera.

A standard means to identify food sources is the direct analysis of gut contents or of termite tissue [11, 12]; yet, the small amount and heterogeneity of gut material made this approach less practicable. Another approach is not to study the termite itself, but to analyze the potential food source and the material used to build their nests [13, 14]. For the most termite genera they comprise to a large degree of termite feces [15, 16], finely distributed in the nest and integrating across all past food sources that the termites digested. In a previous work in the rainforests nearby Manaus, Brazil, various nest types and potential food sources of different termite genera had been screened for their contents of C, N, lignin, and heavy soil minerals in order to elucidate the feeding guild [17]. The results indicated that termite nests were significantly enriched in soil organic matter (SOM) relative to the surrounding soil. It was proposed that *Nasutitermes* sp. [18] and *Cornitermes* sp. [19] belong to the wood-feeders, *Termes* sp. [18, 19], *Embiratermes* sp. [18], and *Anoplotermes* sp. [19] to the soil/wood-interface feeders, and that *Constrictotermes* sp. probably uses microepiphytes as food source. This classification was made according to the recent classification [14], the degree of SOM enrichment in the nests being dependent on the feeding guild. In this regard, soil/wood interface feeder exhibited lower C contents but more heavy fraction SOM in their nests than xylophagous (wood-feeding) termites, for instance, which significantly accumulated the light fraction of SOM. However, separating termite nests into density fractions, followed by lignin analysis, is time consuming. Moreover, it does not provide information on possible microbial alteration of the food source material within the nest after construction. Such information, however, may be needed when microbial ‘fingerprints’ might influence the feeding guild classification. Such information may also be helpful in understanding whether microbial alteration of food sources in the nests could also be linked to feeding guild. With the advances of analytical technical development, fast and reliable methodologies are now available to identify both food sources from candidate substrates as well as the origin and subsequent processing of organic matter in nests. These techniques could thus provide an independent and even more rapid assessment of feeding guilds than achieved before by time-consuming density fractionation and lignin tracing.

As a first step, the used food source has to be unequivocally identified. For this, stable carbon (C) isotope analysis of termite body tissue and termite modified material has been successfully applied in the past for various ecosystems [20–22]. Using this approach it is possible to characterize the parent plants as C3 (stable isotope ratios -21 to -33‰) or as C4 (ratios are between -9 to -17‰) [23]. When consumed by animals, the stable C isotope composition of the food source is not significantly altered [24] and, thus, the stable isotope ratios of the termite nests and mounds indicate the type of plants consumed (C3 or C4 plants). However, stable C isotope analysis might be of limited use due to the dominance of C3 plants in the rainforests. Stable nitrogen (N) isotopes, however, might give a more detailed insight into termite nutrition, as it was found that soil-feeders are more enriched in ^15^N than wood-feeders [13, 22, 25], which might also be reflected in the nest material. Using δ^14^N values it was even possible to show that sympatric soldierless soil-feeding neotropical rainforest termites feed on distinct components of the soil. In addition the δ^15^N values indicated that some termite species exhibited a more pronounced resource partitioning than others, reflecting differences in habitat and niche conditions and presence of competitors [26].

Polysaccharide analyses are also promising for elucidating the chemistry of termite nests and relating it to the food source and niche conditions. Microorganisms mainly metabolize hexoses and, with this, synthesize pentoses [27, 28]. Similarly, the ratio between these non-cellulosic polysaccharides (NCPS), originating from both plants and microbes, and cellulosic polysaccharides (CPS), which only occur in plant cells, provide a hint on polysaccharide origin and the intensity of microorganism impact to the SOM in a given soil environment [29, 30]. When combined with amino sugar analysis, we may also trace the microbial origin of soil organic N [31]. This approach is based on the observation that muramic acid (MurAc) uniquely originates from bacteria cell walls, whereas glucosamine (GlcN) is an important constituent of fungal chitin. Different ratios among amino sugars may thus be used to characterize the residues of the microbial community structure [32–34] in the termite nests and potential food sources.

Compared with the biomarker analysis mentioned above, pyrolysis-field ionization mass spectrometry (Py-FIMS) is a powerful analytical tool for the characterization of the overall molecular composition of SOM [35]. The mass spectra obtained provide molecular information about the origin of the samples and thermograms obtained simultaneously reveal the strength of the chemical bonding within organic molecules, or between the SOM and mineral particles [36]. This method can give detailed insights into the organic matter composition of the potential food source and also of the termite nest material, and, therefore, may enable transformation processes to be characterized. The aim of this study was to confirm the hypothesis that according to the termite nest chemistry both food sources and their microbial alteration of the Amazonian termites with known feeding guilds can be identified, even when also microbial alteration processes in these nests are considered.

## Materials and Methods

### Samples

All of the samples were collected in the Amazon region (Terra Firme) in Manau. The natural vegetation in the study area is lowland tropical rainforest mixed with fallow land, agricultural land, and secondary forests [37]. The permission for the soil sampling campaign was granted by Embrapa Amazônia Ocidental for all sampling locations. The contact person is Marcos V.B. Garcia (mgarcia@cpaa.embrapa.br). The coordinates of the sampling position were 02°59'S latitude and 59°59'W longitude, having a mean annual precipitation of 2100 mm with a maximum of 200 to 300 mm per month, and a mean annual temperature of 25°C [38], and a dry season generally lasting for 2 month. The sampled soil was a Xanthic Ferralsol. All soil characteristics were determined using standard methods as outlined by [39] and the soil had a total nitrogen content of 0.22% ± 0.08, sulfur content of 0.04% ± 0.01, a pH value of 3.8 (H_2_O), and a cation exchange capacity of 11.2 ± 8 mmol_c_ kg^-1^. The same sample set was already analyzed for their phosphorus forms [39], they were screened for polycyclic aromatic hydrocarbons [40], and characterized for their lignin signature [17].

Samples were taken from two different nest of each genera and species studied. Composite samples were taken from different nest parts of intact and inhabited nests (outer wall, inner wall, and central part) belonging to different termite genera and species (Table 1). However, it was not possible to separate the nests into discernible parts for every termite species. Termite mounds were sampled 5, 20 and 35cm above the ground level if available. Nests that were not occupied by termites were discarded, because we were afraid that nest properties changed after abandonment of the nest by the nest-building species (see also [39]). Beside termite nests, we also took composite samples of the surrounding soils and plants, which are possible food sources of the wood, microepiphytes, and soil/wood interface-feeding genera studied, were also obtained. Topsoil samples were taken at 0–10 cm, after removing the organic O layer, using a core sampler. Topsoil samples corresponded to each nest at five subsites located in a radius of about 3–5 m, which were pooled to one sample per nest. The wood samples comprised fresh and partly decomposed stem material, twigs, and bark of the dominating species adjacent to the termite nests. The identified tree species consisted of *Attalea attaleoides* (Barb. Rodr.)Wess. Boer., Arecaceae; *Rinorea racemosa* (Mart.) Kuntze, Violaceae; *Psychotria medusula* Müll. Arg., Rubiaceae; *Naucleopsis ternostroemiiflora* (Hildbr.) C.C.Berg, Moraceae; *Swartzia cuspidata* Benth., Fabaceae; *Swartzia ulei* Harms, Fabaceae; *Ocotea floribunda* (Sw.) Mez, Lauraceae; *Socratea exorrhiza* (Mart.) H. Wendl., Arecaceae; *Myrcia* sp., Myrtaceae; and *Pourouma ferruginea* Standl., Cecropiaceae. Microepiphytes samples were carefully separated from the bark of three standing trees. In the one case where direct feeding of *Constrictotermes* on these microepiphytes could be observed [9]. Due to the very limited samples obtained only C, N, and Py-FIMS could be recorded for these microepiphytes samples. All samples were immediately air dried and sieved to < 2 mm for further analysis.

**Table 1:** Feeding habit, concentrations, and standard deviations of carbon (C), the C:N ratios, lignin derived phenols (VSC), δ^13^C and δ^15^N, non-cellulosic polysaccharides (NCPS), cellulosic polysaccharides (CPS), NCPS:CPS ratios, and ratios of GlcN:GalN, Glc:MurAc, GlcN:MurAc, and GalN:MurAc for nest material of different termite genera and their potential food source.

Sample ^a)^	Feeding habit	C ^a)^	C:N	VSC	δ^13^C	δ^15^N	NCPS	CPS	NCPS:CPS	GlcN:GalN	GlcN:MurAc	GalN:MurAc
		(g kg^-1^)		(g kg^-1^ C)	(‰)	(‰)	(g kg^-1^ C)	(g kg^-1^ C)				
Nest of termite genera												
*Nasutitermes* sp.	Wood-feeders	495^a^ ± 14	51^a^ ± 13	225^a^ ± 23	-27.6^a^ ± 1.0	3.0^a^ ± 1.5	163^a^ ± 3	215^a^ ± 5	0.77^a^ ± 0.02	6.9^a^ ± 2.6	14.5^a^ ± 2.1	2.1^a^ ± 0.5
*Cornitermes* sp.	Wood-feeders	384^b^ ± 21	22^b^ ± 2	85^b^ ± 8	-27.6^ab^ ± 0.5	2.2^ab^ ± 1.3	162^ab^ ± 2	228^ab^ ± 20	0.53^b^ ± 0.06	6.2^ab^ ± 1.8	14.7^ab^ ± 3.4	2.4^ab^ ± 1.2
*Constrictotermes* sp.	Microepiphyte-feeders	303^c^ ± 31	18^c^ ± 1	13^c^ ±1	-30.8^c^ ± 0.9	6.0^c^ ± 0.7	167^abc^ ± 56	101^c^ ± 22	1.64^c^ ± 0.21	4.3c± 1.1	14.4^abc^ ± 3.8	3.4^abc^ ± 2.1
*Termes* sp.	Soil/wood-interface feeders	234^cd^ ± 45	27^d^ ± 3	112^e^ ± 11	-28.1^abd^ ± 0.9	2.0^bd^ ± 0.0	135^cd^ ± 8	214^abd^ ± 7	0.65^abd^ ± 0.07	10.2^d^ ± 2.1	9.3^d^ ± 0.2	0.9^d^ ± 0.2
*Embiratermes* sp.	Soil/wood-interface feeders	195^e^ ± 2	18^ce^ ± 0	68^f^ ± 6	-27.5^abde^ ± 0.9	3.1^abde^ ± 1.6	187^ce^ ± 17	187^abe^ ± 44	1.02^e^ ± 0.15	4.1^ce^ ± 0.3	18.0^abce^ ± 5.8	4.3^ce^ ± 1.6
*Anoplotermes* sp.	Soil/wood-interface feeders	168^ef^ ± 21	16^cf^ ± 1	43^g^ ± 7	-28.4^abdf^ ± 0.1	4.5^acef^ ± 1.5	167^abcef^ ± 3	140^f^ ± 28	1.26^ef^ ± 0.28	5.0^bcf^ ± 0.3	24.4^f^ ± 2.1	4.9^ef^ ± 0.2
Potential food source												
Wood	-	476^ag^ ± 6	94^g^ ± 33	124^eh^ ± 10	-29.1^abcdefg^ ± 2.7	3.4^abefg^ ± 1.0	176^abcdefg^ ± 40	637^g^ ± 175	0.28^g^ ± 0.05	2.5^g^ ± 1.2	5.1^g^ ± 3.2	2.0^abcg^ ± 1.4
Microepiphytes	-	461^agh^ ± 16	28^dh^ ± 10	33^gi^ ± 11	n.a.^b)^	n.a.	n.a.	n.a.	n.a.	n.a.	n.a.	n.a.
Soil	-	23^i^ ± 10	12^i^ ± 2	22^j^ ± 3	-27.4^abdeg^ ± 0.4	6.6^c^ ± 0.8	191^ceg^ ± 24	117^cfg^ ± 38	1.78^h^ ± 0.56	2.6^g^ ± 0.6	16.6^abce^ ± 2.1	6.4^h^ ± 1.6

Values followed by the same letters within a column are not significantly different (P < 0.05).

^a)^ data from Amelung et al. 2002,

^b)^ n.a. = not analyzed (all sample material was used for the Py-FIMS analysis); glucosamine (GlcN), mannosamine (ManN), galactosamine (GalN), muramic acid (MurAc)n = 2 for nests, n = 6 for soil and wood samples.

### Chemical analyses

The organic carbon (C_org_) and N concentrations were determined in dried samples of termite nests, soil, microepiphytes, and wood using an Elementar Vario EL C/H/N/S autoanalyzer system. The content of lignin-derived phenols (VSC) was determined using alkaline CuO oxidation according to the procedure proposed in [41] as modified in [42] and [30].

For carbohydrate analysis, the samples of the nest material and the soil and plant samples were sequentially hydrolyzed as described in [30]. In short, NCPS were hydrolyzed with 1 *M* HCl at 100°C for 5 h [43] (modified). The CPS of the residues were digested using 12 *M* H_2_SO_4_ [44]. The digest was analyzed colorimetrically for carbohydrates as described in [45].

Amino sugars and MurAc were determined according to the description in [28]. In short, the samples were hydrolyzed for 8 h using 6 *M* HCl at 105°C and then purified using 0.5 *M* KOH at a pH between 6.6 to 6.8. The simultaneous analysis of amino sugars and MurAc was done by gas chromatography and followed the derivatization described in [32]. Total amino sugar contents were calculated as the sum of the four amino sugars GlcN, galactosamine (GalN), mannosamine (ManN), and MurAc.

The experimental set up for Py-FIMS has been described in detail in [36]. For temperature-resolved Py-FIMS, about 0.5 mg of the samples were heated in a vacuum of 10^−4^ Pa from 110°C to 700°C, in temperature steps of 10°C over a time period of 15 minutes with a direct inlet probe on the double-focusing mass spectrometer (Finnigan MAT 731, Germany). Between magnetic scans the emitter was flash heated to remove residues of pyrolysis products. During the analysis, 60 spectra were recorded in the mass range *m/z* 15…900. For each sample, three replicates were measured and the data averaged. Thermograms were obtained by plotting the total ion intensities (TII) normalized to sample weight against the pyrolysis temperature. The averaged (replicate measurements) and summed (over the whole temperature range) Py-FI mass spectra are calculated and plotted. For the interpretation we used marker signals (*m/z*) that are assigned to relevant compound classes as described in [36, 46–48].

### Isotope analysis

Termite nest, soil, and wood samples were combusted to CO_2_ for mass spectrometric analysis of ^13^C/^12^C by isotope ratio mass spectrometry (EA-IRMS, Thermo Finnigan MAT, Bremen, Germany) via a Conflow II interface (Thermo Finnigan MAT, Bremen, Germany). Sucrose (ANU, IAEA, Vienna, Austria), CaCO_3_ (NBS 19, Gaithersburg, USA), and ammonium sulphate (N1 and N2, both IAES, Vienna, Austria) were used as calibration standards [49]. From these analyses isotope ratios of ^13^C/^12^C and ^15^N/^14^N were determined expressed in “delta” notation (δ^13^C and δ^15^N) as:

$$\delta \;‰\;=\;\left(\frac{R_{sample}}{R_{standard}}-1\right)\times 1000$$

where, R_sample_ and R_standard_ are the ratios ^13^C/^12^C or ^15^N/^14^N of the sample and standard, respectively. The ratios ^13^C/^12^C or ^15^N/^14^N of the sample and standard, respectively were expressed relative to the international standards Pee Dee Belemnite (PDB) (Vienna Pee Dee Belemnite for carbon) and air (atmospheric N_2_), respectively. All analyses were performed at the Institute of Soil Science and Soil Geography, University of Bayreuth.

### Statistical analyses

To test the differences between feeding guilds, all data was statistically analyzed with IBM SPSS 20 [50] using an analysis of variance (ANOVA) with a post hoc Tukey test. The significance level was set at P < 0.05. Non-metric multidimentional scaling (NMDS) was used to identify similarities among the termite nest samples and the food source soil and wood samples, in terms of the ratio between NCPS and CPS, the ratio between GalN and MurAc, δ^15^N, δ^13^C values, and total amino sugar content. Additionally, a second NMDS was performed on the Py-FIMS mass spectra as the potential food source microepiphytes were only analyzed using this method. The NMDS analyses were done in R (version 3.0.2) using the Vegan package for R [51]. A two-dimensional ordination was achieved using the Bray-Curtis dissimilarity index. Linear regression analysis was performed to elucidate relationships between the concentration of lignin monomers as determined by PyFIMS and that of lignin derived phenols reported in [17].

## Results

### Isotope analysis

When comparing the δ^13^C values of termite nests samples and potential food sources, significant differences for isotope values were identified on the basis of non-overlapping standard deviations. The δ^13^C value of the nest material of the soil/wood-interface feeders tended to increase in the order *Anoplotermes* ≤ *Termes* ≤ *Embiratermes* = soil, but differences were not significant and the values were thus not significantly different within the ranges found for the wood-feeders *Nasutitermes* and *Cornitermes* (Table 1). The δ^13^C value of the wood sample was lower compared to the soil sample (-29.1‰ vs. -27.4‰), and only in tendency also lower than for the wood-feeders. Considering the large variation of δ^13^C values in wood (± 2.7‰), there was no overall difference from the isotopic signature of the nests of the wood feeders. The δ^13^C value of the nest material of *Constrictotermes* was lower compared to all other samples analyzed having a value of -30.8‰, but interpretation is difficult due to the lack of values for microepiphytes (Table 1).

The δ^15^N value of the nest material of the soil/wood-interface feeder only partly reflected the differences in δ^13^C values, and δ^15^N increased in the order *Termes* ≤ *Embiratermes* ≤ *Anoplotermes* and among the wood-feeders *Cornitermes* ≤ *Nasutitermes* (Table 1). The δ^15^N value of the wood sample was again lower than that for the soil sample (3.4‰ vs. 6.6‰). The δ^15^N value of the nest material of *Constrictotermes* was exceptionally higher than that of all other samples from termite nests (Table 1).

### Polysaccharides

Polysaccharides can be used as both carbon and energy source, i.e., they are prone to microbial transformation. We determined the content of NCPS and CPS and used the quotient of these values as an approximation for the degree of microbial SOM alterations by the various termite genera. The NCPS contents in the termite nest material decreased in the order *Termes < Anoplotermes < Embiratermes <* soil for the soil/wood-interface feeders, and it was similar for the *Nasutitermes* and *Cornitermes* genera nest samples, which however exhibited lower NCPS contents than the pure wood. The order of CPS contents was the opposite, although the wood again contained higher CPS contents than the nests of the wood-feeders. Apparently, polysaccharides were lost during nest construction of the wood-feeders.

Calculating the ratios of NCPS to CPS revealed that ratios of *Embiratermes* and *Anoplotermes* were comparable to the ratio of the soil sample, whereas NCPS:CPS was lower in nest samples of *Termes*, *Cornitermes*, and *Nasutitermes* with the wood sample exhibiting the lowest ratio and, thus, the lowest degree of microbial polysaccharide alteration (Table 1). Among all nest samples, the CPS content of *Constrictotermes* was lowest and NCPS:CPS ratio was highest. This gives rise to the question whether SOM was particularly altered by microorganisms in the nest material of *Constrictotermes*, being further examined by amino sugar analysis.

### Amino sugars

Amino sugars are markers for microbial residues in soil. Their concentration in the nest samples and the potential food sources ranged between 3 (wood sample) to 30 mg kg^-1^ TOC (soil sample), i.e., both major food sources comprised borders of low and high accumulation of microbial products among all samples, respectively. Thus, as a tendency, the wood-feeders had lower amino sugar concentrations than the soil/wood-interface feeders, with differences between the guilds (*Nasutitermes < Cornitermes*, and *Termes* < *Embiratermes* < *Anoplotermes)*. *Constrictotermes* showed amino sugar concentrations in the range of the wood-feeders and SOM in its nest material therefore seems not particularly altered by microorganisms.

**Fig 1:**
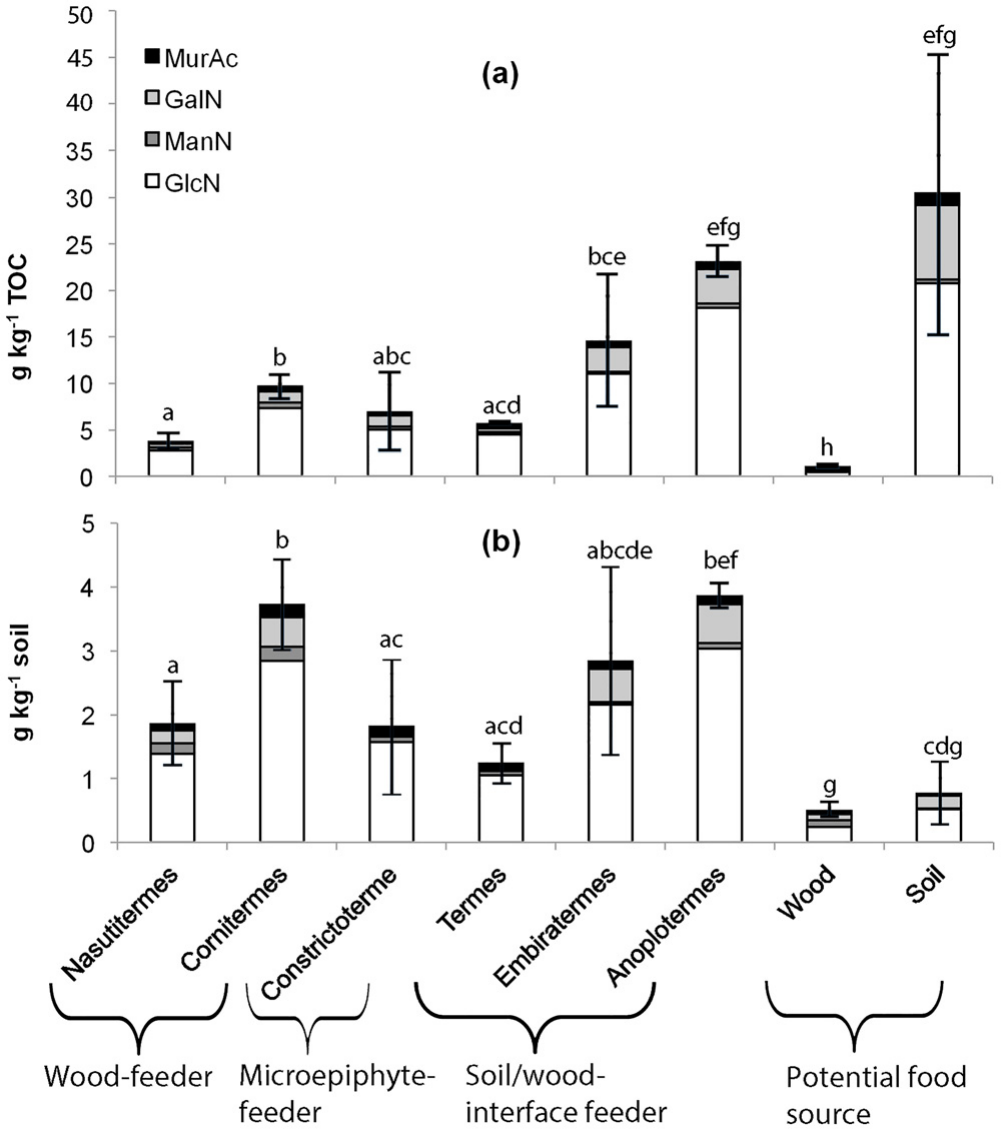
Amino sugars (galactosamine = GalN, mannosamine = ManN, glucosamine = GlcN) plus muramic acid (MurAc) in nest samples of different termite genera and potential food sources (a) referred to total organic C and (b) referred to soil weight. The standard deviation (SD) belongs to the sum of all amino sugars analyzed. Sum of amino sugar followed by the same letter were not significantly different (P < 0.05).

Glucosamine contributed most to the total amino sugar content found, comprising on average 75% of the microbial residues in nest and soil samples, and 49% of those in the wood samples (Fig 1). Hence, the portion of GlcN derived primarily from chitin from fungal cell walls, and to a minor degree also from termites, arthropods and other sources, was largest in soil and smallest in wood, even if the wood and microepiphyte-feeders did not show consistently lower GlcN:MurAc ratios than the soil/wood-interface feeders (Table 1). The latter, however, showed a clear differentiation according to their GalN:MurAc ratio (Table 1). In order to identify the influence of microorganisms and fungi on material transformations, detailed knowledge about the nest chemistry is essential, which is provided by Py-FIMS analysis.

### Pyrolysis-field ionization mass spectrometry (PY-FIMS)

**Fig 2:**
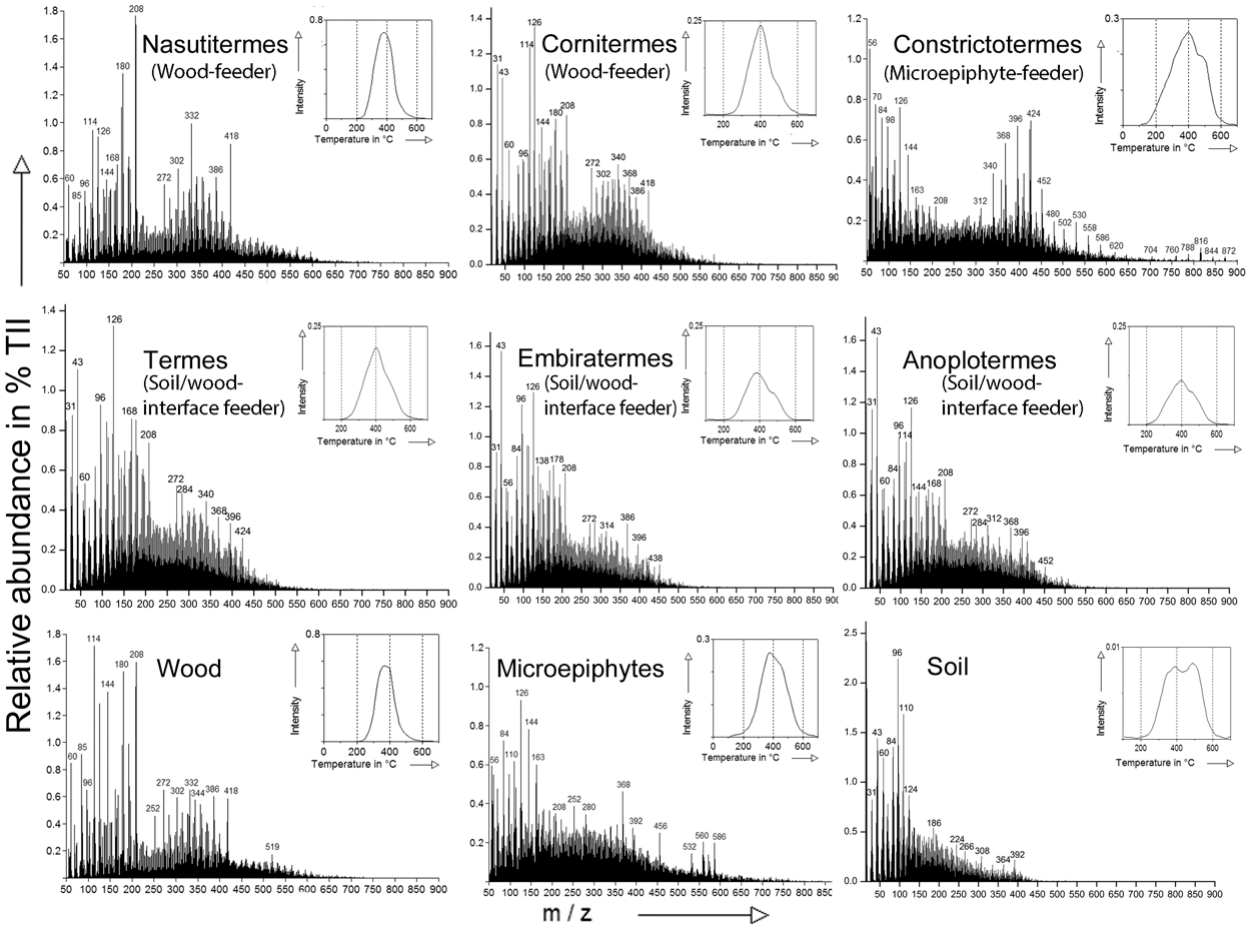
Thermograms of total ion intensity (TII) (upper right) and summed averaged pyrolysis-field mass spectra of samples of nest material from different termite genera and species and their food sources.

The Py-FIMS spectra give an overview about relevant molecules contained in termite nests and food sources. The molecules are released at increasing pyrolysis temperature, i.e., the thermograms give additional information on the stability of the compound bondings against heating. Here, the signal patterns of the summed Py-FI mass spectra were similar in shape for wood and nests of the two wood-feeders *Nasutitermes* and *Cornitermes* (Fig 2). They exhibited characteristic fragments for lignin monomer units of coniferyl alcohol (*m/z* 180), sinapyl aldehyde (*m/z* 208), pentose (*m/z* 114), hexose (*m/z* 126), other carbohydrates (*m/z* 60, 85, 96, 114, 163), and lignin dimers (*m/z* 272, 302, 332, 344, 386, 418). The shape of the summed Py-FI mass spectrum of the soil sample fitted the shape of the nest material of *Termes*, *Embiratermes*, and *Anoplotermes* (Fig 2) with characteristic fragments of carbohydrates (*m/z* 60, 96, 126) as well as lignin monomer units (*m/z* 110, 124). However, a higher relative abundance of *m/z* 100…200 was present in termite nest samples, being fragments of pentose (*m/z* 114), hexose (*m/z* 126), lipid dimer units (*m/z* 272, 284, 312, 314, 340), and saturated n-fatty acid units (*m/z* 368, 396, 424, 438, 452). In the Py-FI mass spectra of microepiphytes and *Constrictotermes* nest samples, we found dominating mass fragments of carbohydrates (*m/z* 84, 98, 110, 126, 144) and lignin monomer units (*m/z* 164, 208). The spectra differed with respect to a higher relative abundance for *m/z* > 350 in the spectrum of the nest material of *Constrictotermes* (Fig 2), which can be assigned to saturated n-fatty acid units (m/z 340, 368, 396, 424, 452, 480), suberins (*m/z* 502, 530), and n-alkyl esters (*m/z* 620, 704).

When comparing the Py-FI mass spectra for the potential food sources, the wood sample revealed the highest total ion intensity (TII) and the soil sample the lowest (Table 2). Assignment of marker signals to important compound classes of SOM revealed that the proportion of lignin derived phenols where highest among all compound classes. The proportions of phenols and lignin monomers (PHLM; Table 2) significantly correlated with the concentration of lignin derived phenols (VSC; Table 1) (r = 0.61; *P* < 0.01) and, thus, the reliability of the methods was confirmed. Besides, we found significantly lower proportions of long-chained hydrocarbons, sterols, suberin, and free fatty acids in the soil sample compared to other food sources. In contrast, N-containing compounds like peptides as well as non-peptidic compounds like N-containing heterocycles were significantly enriched in the soil. The nest material of *Constrictotermes* was depleted in the proportions of phenols, lignin monomers, and other alkylaromatics, whereas the free fatty acid proportion was elevated (Table 2).

**Table 2:** Total ion intensity (TII) and proportions of ion intensity of different compound classes for nest material of different termite genera and their potential food source.

Samples	TII	%TII from compound classes ^a)^										
	(10^6^ counts mg^-1^)	CHYDR	PHLM	LDIM	LIPID	ALKYL	NCOMP	STEROL	PEPTI	SUBER	FATTY	*m/z* 15…56
**Nest of termite genera**												
*Nasutitermes* sp.	10.7^a^ ± 2.3	6.5^a^ ± 0.7	14.1^a^ ± 0.7	1.7^a^ ± 0.1	4.6^a^ ± 0.1	9.4^a^ ± 0.3	1.1^a^ ± 0.0	4.8^a^ ± 0.1	3.2^a^ ± 0.2	1.0^a^ ± 0.2	1.5^a^ ± 0.2	4.4^a^ ± 1.0
*Cornitermes* sp.	3.6^b^ ± 1.6	8.5^ab^ ± 1.1	11.3^b^ ± 1.0	2.1^a^ ± 0.3	4.5^ab^ ± 0.2	8.5^b^ ± 0.1	2.1^b^ ± 0.1	3.9^ab^ ± 0.6	5.0^b^ ± 0.2	0.7^ab^ ± 0.2	1.0^b^ ± 0.1	6.0^ab^ ± 0.5
*Constrictotermes* sp.	6.5^abc^ ± 3.1	6.3^ac^ ± 1.3	5.2^c^ ± 0.3	1.5^a^ ± 0.4	2.8^c^ ± 0.3	5.0^c^ ± 0.2	2.4^bc^ ± 0.2	4.6^abc^ ± 0.4	5.9^bc^ ± 0.6	1.0^abc^ ± 0.3	2.9^c^ ± 0.8	15.5^c^ ± 4.0
*Termes* sp.	3.2^bd^ ± 1.0	8.6^bcd^ ± 0.4	12.9^abd^ ± 0.3	2.2^a^ ± 0.1	4.1^ad^ ± 0.2	9.0^ab^ ± 0.3	2.8^cd^ ± 0.3	3.0^bd^ ± 0.2	5.7^bcd^ ± 0.4	0.4^bcd^ ± 0.2	1.5^ad^ ± 0.1	8.1^d^ ± 0.5
*Embiratermes* sp.	2.3^bd^ ± 0.3	10.3^bcf^ ± 1.1	12.7^abe^ ± 1.1	1.8^a^ ± 0.1	3.7^de^ ± 0.2	8.6^bd^ ± 0.4	3.2^cde^ ± 0.3	2.5^de^ ± 0.5	6.5^cde^ ± 0.6	0.3^bcde^ ± 0.2	1.5^abc^ ± 0.5	10.1^cde^ ± 0.9
*Anoplotermes* sp.	2.1^bde^ ± 0.7	9.00^bcdf^ ± 0.8	10.5^bef^ ± 0.8	2.0^a^ ± 0.5	3.7^de^ ± 0.3	7.8^bd^ ± 0.6	2.9^cdef^ ± 0.1	3.2^bdef^ ± 0.7	6.4^cdef^ ± 0.4	0.4^bcde^ ± 0.1	1.5^abc^ ± 0.6	10.8^cde^ ± 1.4
**Potential food source**												
Wood	7.9^ac^ ± 1.9	9.4^bcdef^ ± 0.7	12.2^befg^ ± 0.4	1.6^a^ ± 0.2	4.3^abde^ ± 0.3	9.3^abde^ ± 0.5	1.2^ag^ ± 0.1	4.1^abcfg^ ± 0.4	3.7^a^ ± 0.4	0.7^abcdef^ ± 0.1	1.6^a^ ± 0.2	5.0^abf^ ± 1.0
Microepiphytes	5.6^bcf^ ± 0.5	7.3^acdg^ ± 0.6	6.3^dh^ ± 0.2	1.7^a^ ± 0.2	3.1^cef^ ± 0.3	6.6^f^ ± 0.4	2.4^cd^ ± 0.1	2.9^bdef^ ± 0.4	6.2^cdef^ ± 0.4	0.6^abcdef^ ± 0.1	1.4^ab^ ± 0.3	10.0^eg^ ± 0.3
Soil	0.2^g^ ± 0.0	11.3^bcef^ ± 1.3	11.0^befg^ ± 0.4	2.1^a^ ± 0.1	2.8^cf^ ± 0.1	8.2^abde^ ± 0.5	6.3^h^ ± 0.2	1.3^h^ ± 0.3	11.8^g^ ± 0.9	0.1^g^ ± 0.1	0.2^e^ ± 0.1	14.9^c^ ± 2.0

Values followed by the same letters within a column are not significantly different (P < 0.05).

^a)^ CHYDR, carbohydrates; PHLM, phenols and lignin monomers; LDIM, lignin dimers; LIPID, long-chained hydrocarbons; ALKYL, alkylaromatics; NCOMP, N-containing non-peptidic compounds; STEROL, sterols; PEPTI, peptides; SUBER, suberin; FATTY, free fatty acids C_16_-C_34_.

**Fig 3:**
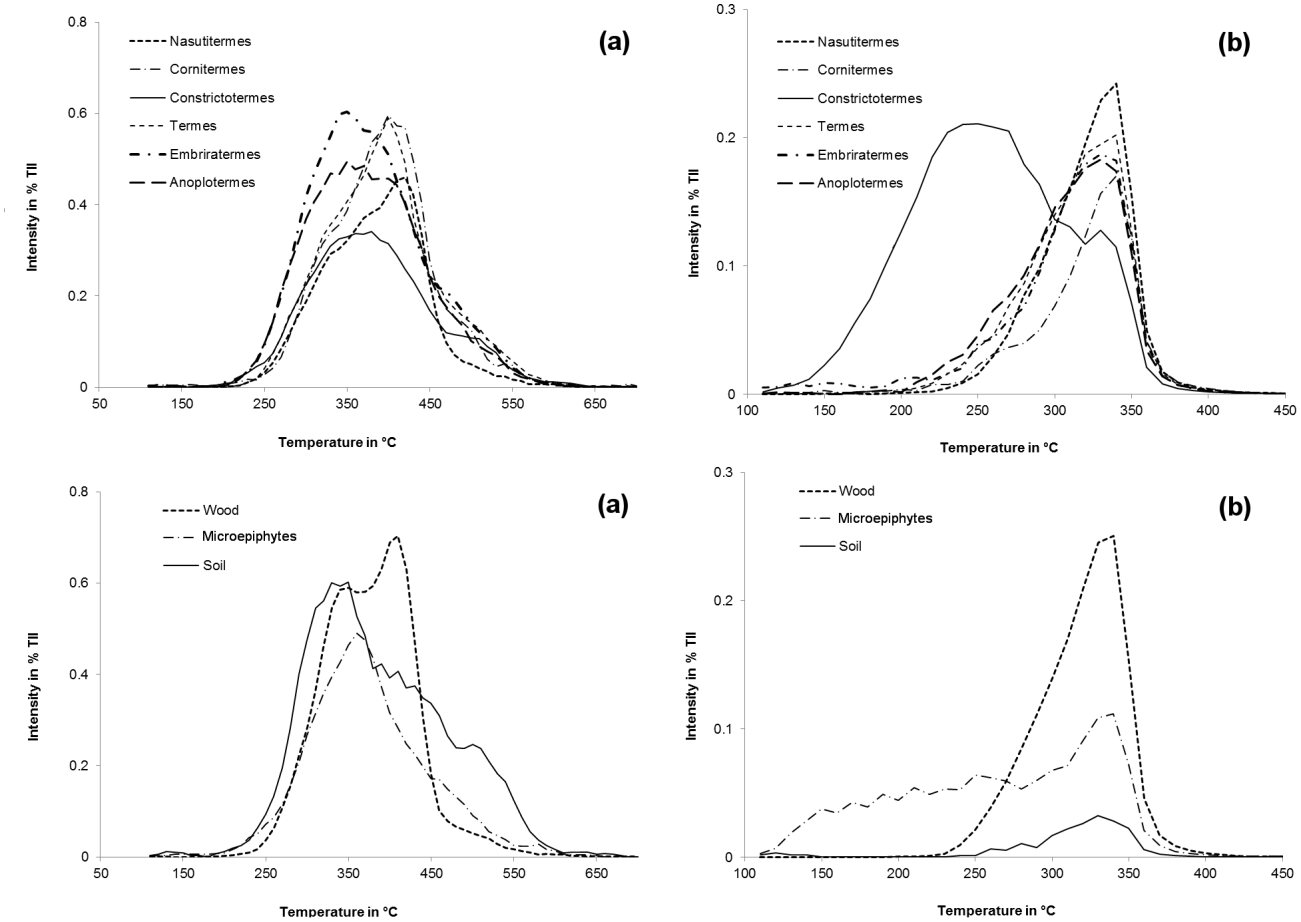
Pyrolysis field thermograms of the compound classes carbohydrates (a) and free fatty acids (b) normalized to the total ion intensity (TII) for nest material of different termite genera and their potential food source.

In Fig 2 the TII thermogram (see upper right inserts) for the soil as well as for the nest samples of *Constrictotermes* and *Embiratermes* were bimodal in shape, whereas the thermogram of the microepiphytes, wood, and samples of the nest material of *Nasutitermes*, *Cornitermes*, and *Termes* were of monomodal shape, though a shoulder was visible in the thermogram of *Cornitermes* and *Termes* around 430°C (Fig 2). Thermograms of the soil/wood-interface feeding guild as well as their respective food source showed a volatilization maximum around 380°C, whereas thermograms of microepiphytes, soil, and nest material of *Constrictotermes* and soil-feeders showed the volatilization maximum around 400°C, with a second temperature maximum slightly visible around 500°C (Fig 2). Hence, the presence of soil shifted the release of molecules to higher temperatures, whereas in the thermogram of the compound class carbohydrates including pentose and hexose units (Fig 3a), the volatilization maximum of the wood sample was shifted to higher temperatures compared to the soil sample (410 *vs*. 350°C). Additionally, the soil sample showed an additional compound volatilization at temperatures ≥ 500°C. The temperature maximum in the nest sample of *Nasutitermes* was around 410 and 420°C and for *Cornitermes* and *Termes* about 400°C, whereas for *Embiratermes* and *Anoplotermes* it was at 350°C. The volatilization maximum of free fatty acids for all samples was around 330 to 340°C, except for the nest sample of *Constrictotermes* having a volatilization maximum at 250°C (Fig 3b). Additionally, the microepiphytes sample showed a compound volatilization at temperatures ≤ 280°C.

### Data integration by non-metric multidimensional scaling

**Fig 4:**
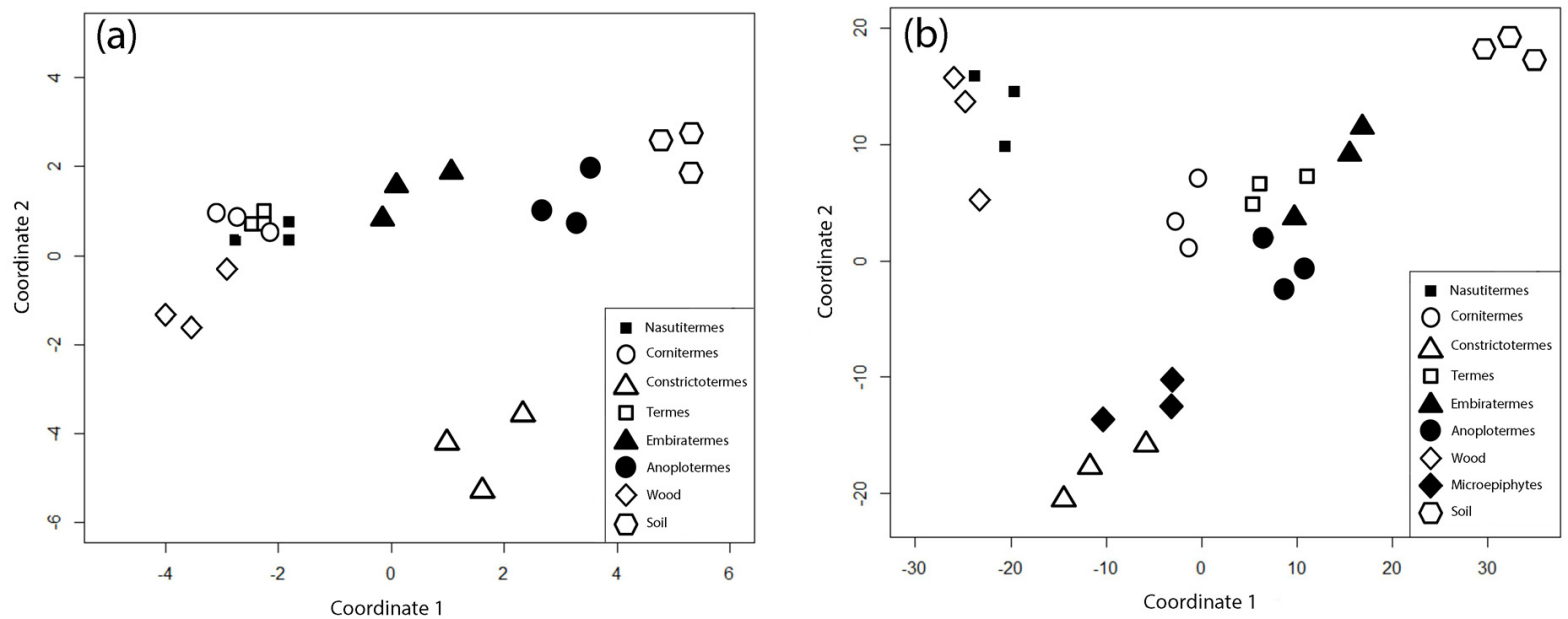
Non-metric multidimensional scaling (NMDS) of samples of nest material from different termite genera and species and their food sources using (a) the non-cellulosic polysaccharides (NCPS) and cellulosic polysaccharides (CPS) ratio, the galactosamine (GalN) and muramic acid (MurAc) ratio, δ^15^N, δ^13^C values, and total amino sugar content as attributes, and (b) pyrolysis-field mass spectra.

In order to disentangle the multiple chemical information from the various methods in relation to feeding guilds of the nest building genera, we performed a NMDS analysis to visualize similarities between the nest material of different termite species and genera and the potential food source (Fig 4). The NMDS analysis using the ratio between NCPS and CPS, the ratio between GalN and MurAc, as well as δ^15^N, δ^13^C values, and total amino sugar content (Fig 4a) resulted in an excellent final Kruskal stress value ─ the degree of correspondence between the distances among points ─ of 8.1x10-14 for a two-dimensional solution [52]. Distances between samples along coordinate 1 were larger compared to coordinate 2. Additionally, distances between nest sample of *Constrictotermes* and the other samples were larger on coordinate 2 compared to coordinate 1. Separation along coordinate 1 was greatest for the wood and soil sample, with all termite nest samples falling between (Fig 4a). Wet chemical data for microepiphytes were lacking due to restrictions in the quantity samples (see above, section 2.1). The MDS analysis using the Py-FI mass spectra (Fig 4b) data resulted in an acceptable final Kruskal stress value of 0.14.

Separation of samples was good for both coordinates. The nest sample of *Nasutitermes* clustered with the wood sample, whereas *Cornitermes* was a little separated from this cluster and closer located to the nest sample of *Termes*, *Embiratermes*, and *Anoplotermes*. The cluster of the soil/wood-interface feeders was almost intermediate between the soil, wood, and microepiphytes samples, but closest located to the soil samples. The nest sample of *Constrictotermes* was most closely located to the microepiphytes and similar to the first MDS analyses shown in Fig 4a clearly separated on coordinate 2 from the other samples (Fig 4b).

## Discussion

### Origin and alteration of organic matter in termite nests

Some chemical properties of the food source may hardly be altered by termites during nest construction using their own feces. Among these properties is the stable carbon isotopic composition [24]. The rainforest trees predominantly have C3 photosynthetic pathways [21], and the low δ^13^C values of the composite wood sample are in a similar range as observed in [53] for fresh wood, decaying wood, and bark. Nests of the wood-feeders *Nasutitermes* and *Cornitermes* were slightly enriched in ^13^C, consistent with the findings described in [24] showing that δ^13^C values of the feces of animals were approximately 1‰ more positive than their diets, due to the respiration of lighter CO_2_. The δ^13^C values of the termite nests of the soil/wood-interface feeders were in the same range as the δ^13^C values of the wood samples and, in tendency, lighter but not heavier than those of the soils, reflecting that wood also significantly contributed to the δ^13^C values even of these feeding guilds. Only *Constrictotermes* showed an extraordinary low δ^13^C value, reflecting that it feeds on other sources like microepiphytes [8, 9].

In contrast to the δ^13^C values, the δ^15^N values across all studied soil/wood-interface feeding termites exceeded those of the wood-feeding genera by 0.7‰. This is in line with findings in [22], who also reported higher average δ^15^N values of soil-feeders than of wood-feeders and grass-harvesters species. Such an enrichment may be explained by immobilization and/or mineralization processes during soil N transformations [54], therewith being an indirect tracer of the food source when feeding on soils with higher degree of N transformation compared with wood samples. The main source of soil N in tropical forests is the cycling through plant growth and litterfall processes of the vegetation [55]. Termites play, as mentioned before, an important role as litter shredders and, thus, they substantially contribute to organic matter breakdown and incorporation of N into soil.

Polysaccharides are another, more direct tracer of microbial transformation alike the N isotope composition. Woody plants have high cellulose contents, and indeed, the wood-feeding species of *Nasutitermes* and *Cornitermes* accumulated more CPS in their nests than the soil feeding species. Yet, the quotient of NCPS:CPS was also lower in the nests of these species relative to the respective NCPS:CPS ratios in the nests of soil/wood-interface feeders (Table 1). We attribute this to the decomposition of the cellulose during or after nest construction. Microorganisms synthesize NCPS and, thus, with increasing decomposition of the cellulose, the quotient of NCPS:CPS increases as well [3, 56]. Therefore, higher NCPS:CPS quotients (Table 1) hint at a decomposition of the cellulose and microbial re-synthesis of NCPS rather than to a selection of materials being low in cellulose content for nest construction.

The termites that are closest to soil-feeding habits, in turn, already consume material with high degree of polysaccharide transformation. Hence, elevated NCPS:CPS quotients were detected, because the plant cell wall is already degraded by microorganisms and fungi in soil and glucose is released in a free form. The latter increased in the order *Termes* < *Embiratermes* < *Anoplotermes*, i.e., in the reverse order as the contents of VSC lignin declines. The saccharide data therewith align with findings in [17] who concluded that the degree of soil feeding increased in the same order *Termes* < *Embiratermes* < *Anoplotermes*. Among all termite genera studied, the highest NCPS:CPS ratio was found in the nests of *Constrictotermes*. Yet, the final proof for the chemical assignment of microepiphytes as food source of *Constrictotermes* remained uncertain, as not enough microepiphytes sample material could be obtained for polysaccharide analysis. Very small sample amounts, however, could be used for Py-FIMS.

The Py-FI mass spectra generally confirmed the results obtained by polysaccharide analysis hinting at wood as food source for *Nasutitermes* and *Cornitermes* and at increasing geophagy for *Anoplotermes*, *Embiratermes*, and *Termes*. The Py-FI mass spectra for nest material of the wood-feeders and the wood sample were fairly similar; solely the lower relative abundance of the mass fragment of pentose (*m/z* 114) in the nests samples of wood-feeders supports the digestion of ligno-cellulose by the termites [57]. In the Py-FI mass spectra of soil/wood-interface feeders, lignin dimers, which are hardly altered during digestion, and saturated n-fatty acid dimers were present. Notably, n-fatty acid was very weak in the soil spectrum. This endorses the results described in [17] claiming that *Termes*, *Embiratermes*, and *Anoplotermes* feed on both, soil and wood. Thus, characteristic wood as well as soil fragments can be found in the Py-FI mass spectra of nest samples of the soil/wood-interface feeding guild, making this method to a powerful tool in termite feeding guild identification.

The significantly lower peptide concentrations found in the nest material of soil-feeders compared to the soil sample hints at a strong mineralization of nitrogen in the gut ([58] as reviewed in [59]). Even if the exact assignment for the nest sample of *Constrictotermes* to microepiphytes as food source was difficult, the characteristic signals for microepiphytes (*m/z* > 500) have been present in the nests, though at slightly lower relative abundance. Possibly there was even a selection of food within the variety of microepiphytes by the termites. In any case, in the spectrum of the nest sample of *Constrictotermes* the relative abundance of saturated n-fatty acids was intense, hinting at an enrichment of fatty acids from microepiphytes.

The bimodal shape of thermograms of *Constrictotermes* and the soil sample is the result of the presence of stabile compounds in these samples. These could be charred materials [60] or any other stable organic matter constituent, which was not detected in the nests of the other termite genera. Vice versa, the higher onset and lower offset temperature of the thermograms of nest material of wood-feeders and the wood sample compared to all other samples indicated the absence of substances with distinctive higher and lower thermal stability. When looking at the thermogram of the free fatty acids it is obvious that *Constrictotermes* is enriching the free fatty acids from the microepiphytes (Fig 3b). Interestingly, in the thermograms of the carbohydrates; the shift of the volatilization maximum to higher temperatures for the wood sample compared to the soil sample (Fig 3a) is a result of the high structural stability of polysaccharides, which are present in a higher amount in wood in the form of cellulose and xylane. As a result, the carbohydrate thermograms of the wood-feeders also showed a higher structural stability, due to higher portions of the latter relative to the soil/wood-interface feeders; yet, as a result of cellulose degradation, the second TII peak at higher temperatures had already declined (Fig 3a). Simultaneously, the TII was higher in *Nasutitermes* nest samples than in wood, hinting at an enrichment of volatile substances in the nest material. In part, these substances possibly reflected microbial products like amino sugars (Fig 1). Other sources might relate to the chemical signaling and defense strategy of this genus—in [40], for instance, a pronounced enrichment of naphthalene in *Nasutitermes* nests was also detected, possibly as a result of an interplay and/or synthesis with associated microorganisms [61], and *Nasutitermes* defense relies on spraying of chemicals which are synthesized in the soldier’s head glands [62].

In comparison, the thermograms of the nests of the soil/wood-interface feeders lacked a second volatilization peak that was still detectable in soil. We assume that this finding indicated a different stabilization of labile organic matter due to the digestion by the termites. This is confirmed by the thermogram of the carbohydrates (Fig 3a) showing no additional volatilization at temperatures higher than 500°C compared to the soil sample. Apparently, the soil samples had a more pronounced stabilization of non-structured sugars such as microbial mucilage adsorbed to minerals, dominated in nests [63]. Plausibly, the thermograms of the soil/wood interface feeding genera therefore reflected both a different stabilization mechanism to that in parent soil, promoted by intestinal passage, and microbial alteration of wood used as an alternative C source. To better understand these findings, we re-evaluate the wet-chemical amino sugar data, providing first insights into microbial fingerprints within the nests.

### Microbial fingerprints in the termite nests

Amino sugars in soil usually originate mainly from microorganisms [31]. Different amino sugars characterize the definite decomposer community [64] and the ratios of amino sugars and MurAc have been used to characterize the contribution of microbial residues to SOM [65]. For example, GlcN gives a hint for the performance of fungi or the accumulation of fungal substances [32, 66], as GlcN derived primarily from the chitin in fungal cell walls and GalN is observed in both bacterial gums [67] and fungi [68, 69]. On the other hand, MurAc is exclusively synthesized by bacteria [68] and, therefore, is the best amino sugar marker for SOM originating from bacteria. The ratio between GlcN and MurAc was used to evaluate the fate of bacterial-derived SOM [65, 70]. While this is true for most soil studies, it is certainly not valid for termite nests, as like all arthropods, termites have an exoskeleton containing chitin [71], which influences the GlcN content in the nest explaining the high GlcN contents found in the soil and nest samples compared to the other amino sugars studied. Therefore, it was recommended to use the GalN:MurAc ratio as alternative indicator for the contribution of different microbial residues to SOM [32].

In the samples studied here, the GlcN:MurAc ratios in the nest materials ranged from 9.3 to 24.4. This is at the tail end of the range commonly observed for soils of temperate [72–74], subtropical [32, 70], and tropical climates [32], where GlcN:MurAc ratios reached values of up to 38, despite that we may assume for the samples of this study that large portions of GlcN originated from the termites. It seems, therefore, reasonable to assume that the non-outlying and fairly low GlcN:MurAc ratios in the termite nests reflected a high abundance of bacterial residues. In soils, such high portions of bacterial residues are to our knowledge typical for subsoil horizons [75, 76], therewith giving rise to the hypothesis that at least the soil-wood interface feeders might have done both, include material from deeper soil layers within their nests, and promote symbiosis with bacteria with an increased microbial density 3 to 24 times higher than the neighboring soil [77].

Compared with MurAc, the origin of GalN is less clear. As outlined above, it is abundant in bacterial gums, but may as well be produced by fungi, so that [33] concluded that the bacterial origin of GalN is certain only if the GlcN:GalN ratio correlates positively with the GlcN:MurAc ratio, which is clearly not the case here (r = -0.11, not significant). Moreover, the ratios of GlcN:GalN exceeded the value of 4, whereas in soils they hardly reach the value of three (see references cited above). In part this may be due to additionally GlcN from termites, even if not so abundant that it significantly altered the GlcN:MurAc ratio; in part, it may reflect additional fungal sources of GalN. The latter is supported by the correlation between the GlcN:MurAc and GalN:MurAc ratio (r = 0.91; *P* < 0.01 for the termite nests, and r = 0.69; *P* < 0.05 when soil and wood are included into this correlation). In these regards, bacterial residues dominated the decomposition of organic matter from wood relative to that from soil, and, in the same line, the wood-feeders *Nasutitermes* and *Cornitermes* showed higher predominance of bacterial residues than the soil/wood-interface feeding *Embiratermes and Anoplotermes*. Only *Termes* showed exceptionally high portions of bacterial residues; however, this genus was that one among the soil/wood-interface feeding genera, which also showed the largest preference for wood.

### Feeding guild assignment

The NMDS analysis enabled a precise assignment of the termite genera to feeding guilds and gradations within based on all analysis results with decreasing xylophagy for *Nasutitermes* < *Cornitermes* and increasing geophagy for *Termes* < *Embiratermes* ≤ *Anoplotermes*. Results of decreasing xylophagy confirm the literature as estimated in [17] that, based on various nest properties, *Cornitermes* feeds to a higher degree on non-wood sources compared to *Nasutitermes*. This fact goes along with the finding that the epi-endogeic species *Cornitermes* ─ building their nests down to ≤ 1 m soil depth ─ mainly feed on plant residues from the forest floor [17]. On the other hand, *Nasutitermes* build their nest on tree trunks in a height between 2 to 20 m and mainly feed on woody material [17]. Noteworthy is to our opinion, that this NMDS result was obtained with all parameters also indicating microbial transformations of organic matter. This gives support to our hypothesis that the feeding guild is a more important driver of the heterogeneity of termite nest chemistry within these rainforests than humification and degradation processes in the nests.

Meaningful were also the results obtained for the nest material of *Constrictotermes*. The Py-FIMS NMDS revealed a great similarity between the nest material and the microepiphytes sample. Therefore, NMDS analysis, the high C, and the low lignin content [17] found in the nest material provide direct evidence that *Constrictotermes* does not only feed on microepiphytes [2, 8], but that these structures at least in part resist for prolonged time in the nests.

In summary, a set of various techniques used in combination was shown to be essential to unambiguously identify the food source of termite species. The results of this study demonstrate that a combination of Py-FI mass spectrometry with amino sugar analysis provides the most robust data for a feeding guild classification, This provides a means of independent verification of the combined use of soil density fractionation and lignin analyses for feeding guild identification, as previously done by [17]. The combination with amino sugar analyses is needed, because we have to discount the possibility that relevant food fingerprints in the nests have not been decomposed. The degree of microbial conversion itself is also a good marker for feeding guild assignment by analyzing the nest material of the termites, as it is always highest in soil and, thus, the microbial signature is reflected also in the nest material.

## Conclusions

Chemical data permitted a clear assignment of the termite nests to the feeding guilds of the mound building species, even allowing for possible microbial alterations of the materials during and after building. The joint evaluation of the data support the hypothesis that the main feeding types of termites have a characteristic nest chemistry. The results of polysaccharide analyses suggested that with decreasing geophagy microbial products of metabolism are increasingly represented in the degree of organic matter alteration in the corresponding nests. Thus the degree of organic matter alteration within nests is determined by food choice and not subsequent humification processes in fecal constructions. Similarly, the thermostability of organic matter in the nests also reflects food selection, i.e., the different proportions of mineral material also incorporated. Nevertheless, also the microorganisms leave their fingerprint, as, e.g., evidenced by significantly lower GalN:MurAc values in the *Termes* nests relative to both main food sources. Overall, the chemical nest composition reflect thus trophic niches of the termite genera, and they allow a differentiation of both, subclasses within the main wood and soil/wood-interface feeding guilds, as well as identification of novel food sources such as microepiphytes, particularly when sophisticated bulk sample-screening tools like Py-FIMS are part of the analyses.
