# Correction: Origin and Alteration of Organic Matter in Termite Mounds from Different Feeding Guilds of the Amazon Rainforests

**DOI:** 10.1371/journal.pone.0132876

**Published:** 2015-07-13

**Authors:** The PLOS ONE Staff

## Notice of Republication

This article was republished on June 23, 2015, to replace an incorrectly published version. Please download this article again to view the correct version. The originally published, uncorrected article and the republished, corrected article are provided here for reference.

## Supporting Information

S1 File Originally published, uncorrected article. (PDF)

S2 File Republished, corrected article. (PDF)
